# Healthy food choices are happy food choices: Evidence from a real life sample using smartphone based assessments

**DOI:** 10.1038/s41598-017-17262-9

**Published:** 2017-12-06

**Authors:** Deborah R. Wahl, Karoline Villinger, Laura M. König, Katrin Ziesemer, Harald T. Schupp, Britta Renner

**Affiliations:** Department of Psychology, University of Konstanz, Konstanz, Germany

## Abstract

Research suggests that “healthy” food choices such as eating fruits and vegetables have not only physical but also mental health benefits and might be a long-term investment in future well-being. This view contrasts with the belief that high-caloric foods taste better, make us happy, and alleviate a negative mood. To provide a more comprehensive assessment of food choice and well-being, we investigated in-the-moment eating happiness by assessing complete, real life dietary behaviour across eight days using smartphone-based ecological momentary assessment. Three main findings emerged: First, of 14 different main food categories, vegetables consumption contributed the largest share to eating happiness measured across eight days. Second, sweets on average provided comparable induced eating happiness to “healthy” food choices such as fruits or vegetables. Third, dinner elicited comparable eating happiness to snacking. These findings are discussed within the “food as health” and “food as well-being” perspectives on eating behaviour.

## Introduction

When it comes to eating, researchers, the media, and policy makers mainly focus on negative aspects of eating behaviour, like restricting certain foods, counting calories, and dieting. Likewise, health intervention efforts, including primary prevention campaigns, typically encourage consumers to trade off the expected enjoyment of hedonic and comfort foods against health benefits^1^. However, research has shown that diets and restrained eating are often counterproductive and may even enhance the risk of long-term weight gain and eating disorders^2,3^. A promising new perspective entails a shift from food as pure nourishment towards a more positive and well-being centred perspective of human eating behaviour^1,4,5^. In this context, Block *et al*.^4^ have advocated a paradigm shift from “food as health” to “food as well-being” (p. 848).

Supporting this perspective of “food as well-being”, recent research suggests that “healthy” food choices, such as eating more fruits and vegetables, have not only physical but also mental health benefits^6,7^ and might be a long-term investment in future well-being^8^. For example, in a nationally representative panel survey of over 12,000 adults from Australia, Mujcic and Oswald^8^ showed that fruit and vegetable consumption predicted increases in happiness, life satisfaction, and well-being over two years. Similarly, using lagged analyses, White and colleagues^9^ showed that fruit and vegetable consumption predicted improvements in positive affect on the subsequent day but not vice versa. Also, cross-sectional evidence reported by Blanchflower *et al*.^10^ shows that eating fruits and vegetables is positively associated with well-being after adjusting for demographic variables including age, sex, or race^11^. Of note, previous research includes a wide range of time lags between actual eating occasion and well-being assessment, ranging from 24 hours^9,12^ to 14 days^6^, to 24 months^8^. Thus, the findings support the notion that fruit and vegetable consumption has beneficial effects on different indicators of well-being, such as happiness or general life satisfaction, across a broad range of time spans.

The contention that healthy food choices such as a higher fruit and vegetable consumption is associated with greater happiness and well-being clearly contrasts with the common belief that in particular high-fat, high-sugar, or high-caloric foods taste better and make us happy while we are eating them. When it comes to eating, people usually have a spontaneous “unhealthy = tasty” association^13^ and assume that chocolate is a better mood booster than an apple. According to this in-the-moment well-being perspective, consumers have to trade off the expected enjoyment of eating against the health costs of eating unhealthy foods^1,4^.

A wealth of research shows that the experience of negative emotions and stress leads to increased consumption in a substantial number of individuals (“emotional eating”) of unhealthy food (“comfort food”)^14–17^. However, this research stream focuses on emotional eating to “smooth” unpleasant experiences in response to stress or negative mood states, and the mood-boosting effect of eating is typically not assessed^18^. One of the few studies testing the effectiveness of comfort food in improving mood showed that the consumption of “unhealthy” comfort food had a mood boosting effect after a negative mood induction but not to a greater extent than non-comfort or neutral food^19^. Hence, even though people may believe that snacking on “unhealthy” foods like ice cream or chocolate provides greater pleasure and psychological benefits, the consumption of “unhealthy” foods might not actually be more psychologically beneficial than other foods.

However, both streams of research have either focused on a single food category (fruit and vegetable consumption), a single type of meal (snacking), or a single eating occasion (after negative/neutral mood induction). Accordingly, it is unknown whether the boosting effect of eating is specific to certain types of food choices and categories or whether eating has a more general boosting effect that is observable after the consumption of both “healthy” and “unhealthy” foods and across eating occasions. Accordingly, in the present study, we investigated the psychological benefits of eating that varied by food categories and meal types by assessing complete dietary behaviour across eight days in real life.

Furthermore, previous research on the impact of eating on well-being tended to rely on retrospective assessments such as food frequency questionnaires^8,10^ and written food diaries^9^. Such retrospective self-report methods rely on the challenging task of accurately estimating average intake or remembering individual eating episodes and may lead to under-reporting food intake, particularly unhealthy food choices such as snacks^7,20^. To avoid memory and bias problems in the present study we used ecological momentary assessment (EMA)^21^ to obtain ecologically valid and comprehensive real life data on eating behaviour and happiness as experienced in-the-moment.

In the present study, we examined the eating happiness and satisfaction experienced in-the-moment, in real time and in real life, using a smartphone based EMA approach. Specifically, healthy participants were asked to record each eating occasion, including main meals and snacks, for eight consecutive days and rate how tasty their meal/snack was, how much they enjoyed it, and how pleased they were with their meal/snack immediately after each eating episode. This intense recording of every eating episode allows assessing eating behaviour on the level of different meal types and food categories to compare experienced eating happiness across meals and categories. Following the two different research streams, we expected on a food category level that not only “unhealthy” foods like sweets would be associated with high experienced eating happiness but also “healthy” food choices such as fruits and vegetables. On a meal type level, we hypothesised that the happiness of meals differs as a function of meal type. According to previous contention, snacking in particular should be accompanied by greater happiness.

## Results

### Eating episodes

Overall, during the study period, a total of 1,044 completed eating episodes were reported (see also Table 1). On average, participants rated their eating happiness with *M* = 77.59 which suggests that overall eating occasions were generally positive. However, experienced eating happiness also varied considerably between eating occasions as indicated by a range from 7.00 to 100.00 and a standard deviation of *SD* = 16.41.

**Table 1:** Descriptive statistics for eating happiness by meal type and food category.

Meal type	*N*	*M* (*SD*)	Sum	Min.	Max.	*M*_cwc_ (*SD*)
Meals	1,044	77.59 (16.41)	81,004	7.00	100.00	—
Breakfast	237	74.28 (16.35)	17,604	25.00	100.00	−3.04 (13.41)
Lunch	203	73.09 (18.99)	14,838	7.00	100.00	−4.59 (16.52)
Afternoon tea	27	82.41 (15.26)	2,225	39.00	100.00	5.49 (13.81)
Dinner	245	81.47 (14.73)	19,959	11.00	100.00	4.09 (13.4)
Snack	332	79.45 (14.94)	26,378	13.33	100.00	1.52 (13.93)
Food category (according to the German Nutrient Database)						
Vegetables	400	77.57 (17.17)	27,995	11.00	100.00	1.16 (15.14)
Fruits	218	78.29 (16.13)	15,659	15.67	100.00	−0.65 (13.21)
Sweets	356	78.93 (15.27)	26,443	13.33	100.00	1.68 (13.74)
Salty extras	16	80.40 (10.35)	1,126	57.67	95.33	−0.07 (8.01)
Pastries	14	78.67 (19.25)	1,023	22.67	95.33	−2.39 (18.26)
Bread	284	75.52 (16.33)	19,407	19.33	100.00	−1.55 (13.46)
Pasta	226	77.89 (16.43)	16,123	22.33	100.00	0.39 (15.93)
Cereals	133	75.05 (16.63)	9,082	29.67	100.00	−3.01 (14.13)
Potatoes	61	80.47 (19.07)	4,426	7.00	100.00	1.91 (16.82)
Dairy products	366	75.46 (16.53)	25,127	22.33	100.00	−1.37 (14.49)
Meat	194	78.26 (16.01)	13,382	22.33	100.00	0.26 (14.19)
Eggs	38	79.22 (16.21)	2,852	36.00	100.00	0.95 (15.2)
Meat substitutes	23	83.62 (11.61)	1,672	59.67	100.00	5.39 (10.44)
Fish	26	71.82 (18.65)	1,580	34.33	98.67	−4.58 (16.84)

Note: Eating happiness ranged from 1 (low) to 100 (high). *M*_cwc_ = person-mean centred average happiness score.

### Food categories and experienced eating happiness

All eating episodes were categorised according to their food category based on the German Nutrient Database (German: Bundeslebensmittelschlüssel), which covers the average nutritional values of approximately 10,000 foods available on the German market and is a validated standard instrument for the assessment of nutritional surveys in Germany. As shown in Table 1, eating happiness differed significantly across all 14 food categories, *F*(13, 2131) = 1.78, *p* = 0.04. On average, experienced eating happiness varied from 71.82 (*SD* = 18.65) for fish to 83.62 (*SD* = 11.61) for meat substitutes. Post hoc analysis, however, did not yield significant differences in experienced eating happiness between food categories, *p* ≥ 0.22. Hence, on average, “unhealthy” food choices such as sweets (*M* = 78.93, *SD* = 15.27) did not differ in experienced happiness from “healthy” food choices such as fruits (*M* = 78.29, *SD* = 16.13) or vegetables (*M* = 77.57, *SD* = 17.17). In addition, an intraclass correlation (ICC) of ρ = 0.22 for happiness indicated that less than a quarter of the observed variation in experienced eating happiness was due to differences between food categories, while 78% of the variation was due to differences within food categories.

**Figure 1:**
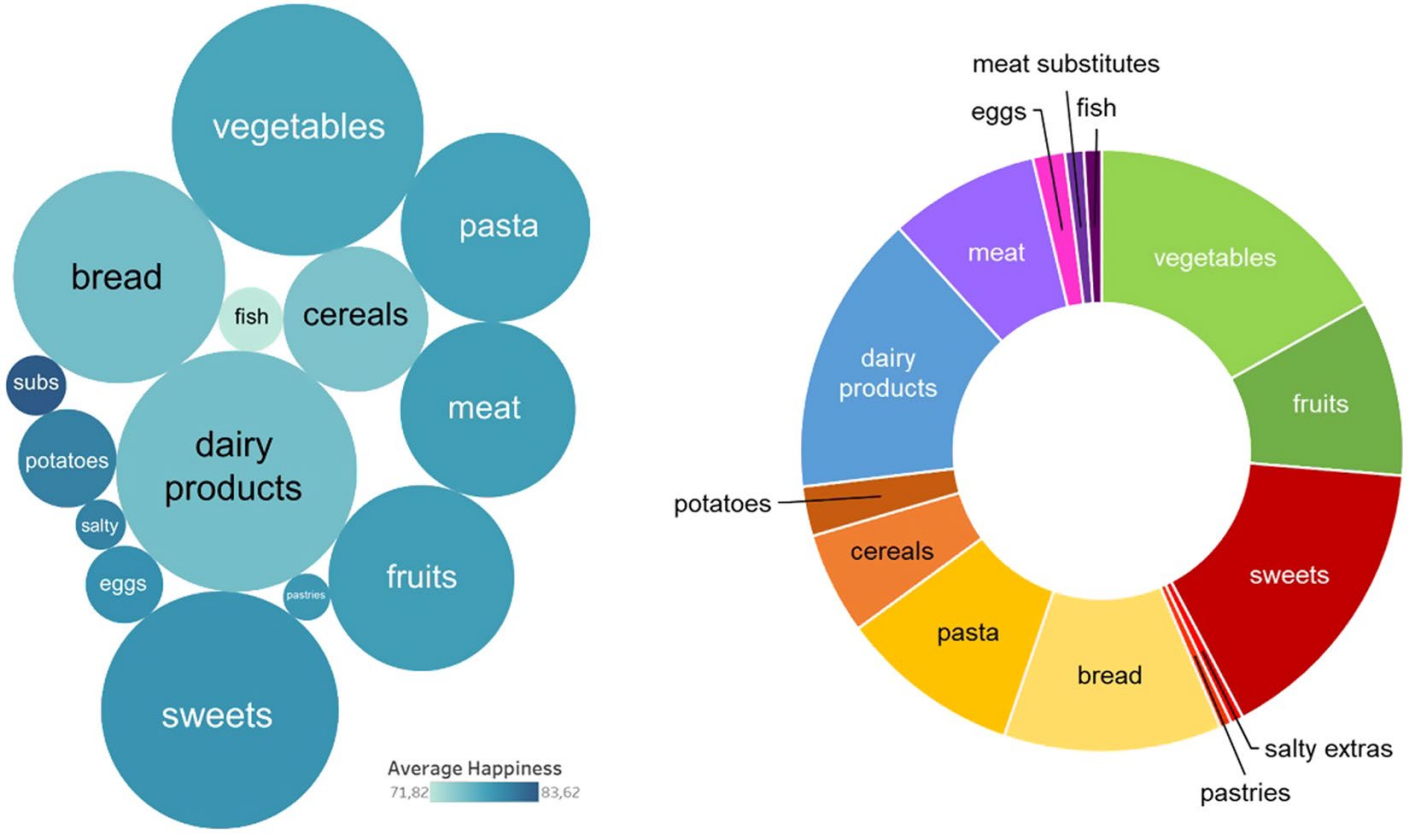
Left side: Average experienced eating happiness (colour intensity: darker colours indicate greater happiness) and consumption frequency (size of the cycle) for the 14 food categories. Right side: Absolute share of the 14 food categories in total experienced eating happiness.

However, as Figure 1 (left side) depicts, consumption frequency differed greatly across food categories. Frequently consumed food categories encompassed vegetables which were consumed at 38% of all eating occasions (*n* = 400), followed by dairy products with 35% (*n* = 366), and sweets with 34% (*n* = 356). Conversely, rarely consumed food categories included meat substitutes, which were consumed in 2.2% of all eating occasions (*n* = 23), salty extras (1.5%, *n* = 16), and pastries (1.3%, *n* = 14).

### Amount of experienced eating happiness by food category

To account for the frequency of consumption, we calculated and scaled the absolute experienced eating happiness according to the total sum score. As shown in Figure 1 (right side), vegetables contributed the biggest share to the total happiness followed by sweets, dairy products, and bread. Clustering food categories shows that fruits and vegetables accounted for nearly one quarter of total eating happiness score and thus, contributed to a large part of eating related happiness. Grain products such as bread, pasta, and cereals, which are main sources of carbohydrates including starch and fibre, were the second main source for eating happiness. However, “unhealthy” snacks including sweets, salty extras, and pastries represented the third biggest source of eating related happiness.

### Experienced eating happiness by meal type

**Figure 2:**
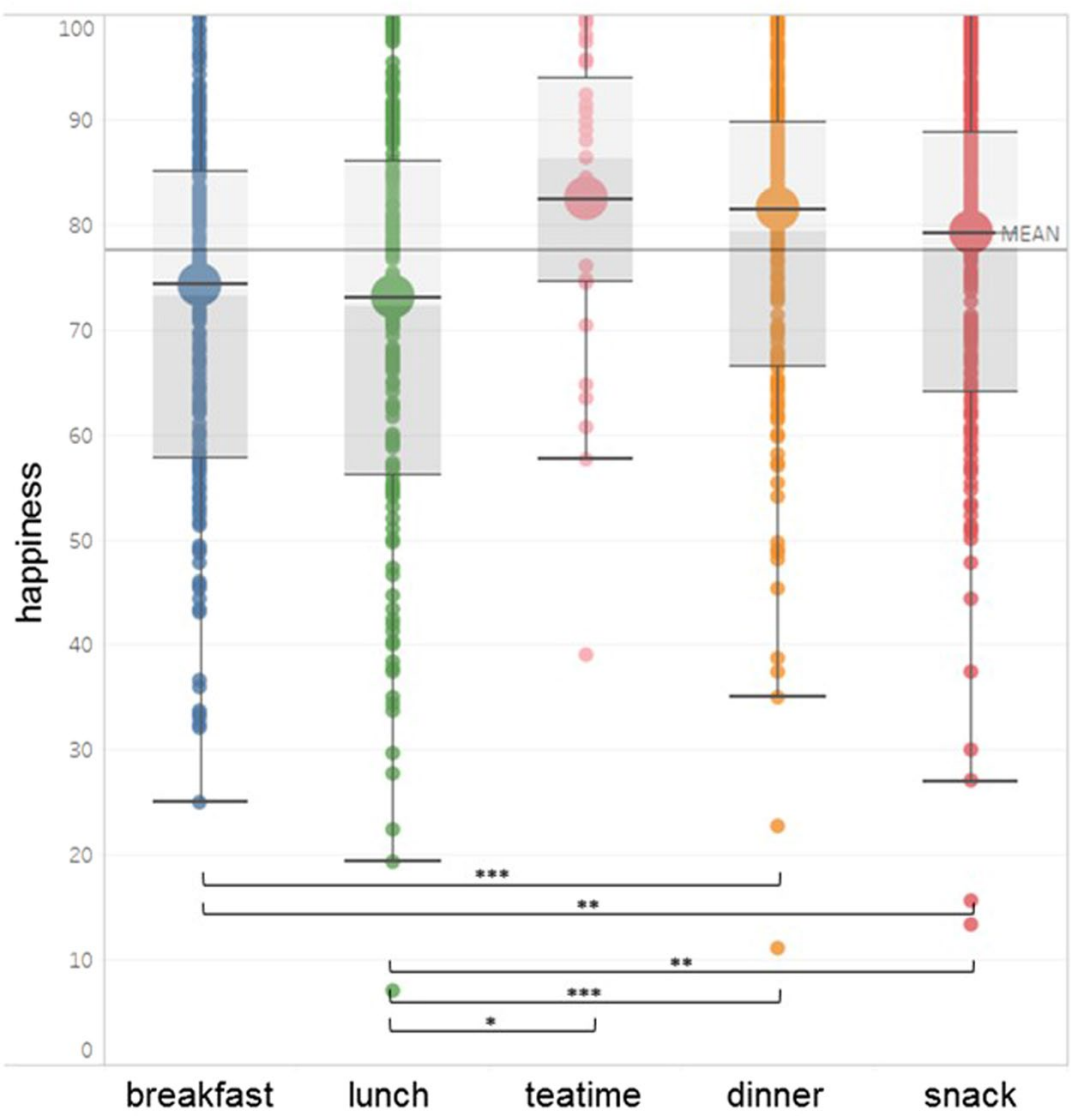
Experienced eating happiness per meal type. Small dots represent single eating events, big circles indicate average eating happiness, and the horizontal line indicates the grand mean. Boxes indicate the middle 50% (interquartile range) and median (darker/lighter shade). The whiskers above and below represent 1.5 of the interquartile range.

To further elucidate the contribution of snacks to eating happiness, analysis on the meal type level was conducted. Experienced in-the-moment eating happiness significantly varied by meal type consumed, *F* (4, 1039) = 11.75, *p* < 0.001. Frequencies of meal type consumption ranged from snacks being the most frequently logged meal type (*n* = 332; see also Table 1) to afternoon tea being the least logged meal type (*n* = 27). Figure 2 illustrates the wide dispersion within as well as between different meal types. Afternoon tea (*M* = 82.41, *SD* = 15.26), dinner (*M* = 81.47, *SD* = 14.73), and snacks (*M* = 79.45, *SD* = 14.94) showed eating happiness values above the grand mean, whereas breakfast (*M* = 74.28, *SD* = 16.35) and lunch (*M* = 73.09, *SD* = 18.99) were below the eating happiness mean. Comparisons between meal types showed that eating happiness for snacks was significantly higher than for lunch *t*(533) = −4.44, *p* = 0.001, *d* = −0.38 and breakfast, *t*(567) = −3.78, *p* = 0.001, *d* = −0.33. However, this was also true for dinner, which induced greater eating happiness than lunch *t*(446) = −5.48, *p* < 0.001, *d* = −0.50 and breakfast, *t*(480) = −4.90, *p* < 0.001, *d* = −0.46. Finally, eating happiness for afternoon tea was greater than for lunch *t*(228) = −2.83, *p* = 0.047, *d* = −0.50. All other comparisons did not reach significance, *t* ≤ 2.49, *p* ≥ 0.093.

### Control Analyses

In order to test for a potential confounding effect between experienced eating happiness, food categories, and meal type, additional control analyses within meal types were conducted. Comparing experienced eating happiness for dinner and lunch suggested that dinner did not trigger a happiness spill-over effect specific to vegetables since the foods consumed at dinner were generally associated with greater happiness than those consumed at other eating occasions (Supplementary Table S1). Moreover, the relative frequency of vegetables consumed at dinner (73%, *n* = 180 out of 245) and at lunch were comparable (69%, *n* = 140 out of 203), indicating that the observed happiness-vegetables link does not seem to be mainly a meal type confounding effect.

Since the present study focuses on “food effects” (Level 1) rather than “person effects” (Level 2), we analysed the data at the food item level. However, participants who were generally overall happier with their eating could have inflated the observed happiness scores for certain food categories. In order to account for person-level effects, happiness scores were person-mean centred and thereby adjusted for mean level differences in happiness. The person-mean centred happiness scores (*M*_*cwc*_) represent the difference between the individual’s average happiness score (across all single in-the-moment happiness scores per food category) and the single happiness scores of the individual within the respective food category. The centred scores indicate whether the single in-the-moment happiness score was above (indicated by positive values) or below (indicated by negative values) the individual person-mean. As Table 1 depicts, the control analyses with centred values yielded highly similar results. Vegetables were again associated on average with more happiness than other food categories (although people might differ in their general eating happiness). An additional conducted ANOVA with person-centred happiness values as dependent variables and food categories as independent variables provided also a highly similar pattern of results. Replicating the previously reported analysis, eating happiness differed significantly across all 14 food categories, *F*(13, 2129) = 1.94, *p* = 0.023, and post hoc analysis did not yield significant differences in experienced eating happiness between food categories, *p* ≥ 0.14. Moreover, fruits and vegetables were associated with high happiness values, and “unhealthy” food choices such as sweets did not differ in experienced happiness from “healthy” food choices such as fruits or vegetables. The only difference between the previous and control analysis was that vegetables (*M*_cwc_ = 1.16, *SD* = 15.14) gained slightly in importance for eating-related happiness, whereas fruits (*M*_cwc_ = −0.65, *SD* = 13.21), salty extras (*M*_cwc_ = −0.07, *SD* = 8.01), and pastries (*M*_cwc_ = −2.39, *SD* = 18.26) became slightly less important.

## Discussion

This study is the first, to our knowledge, that investigated in-the-moment experienced eating happiness in real time and real life using EMA based self-report and imagery covering the complete diversity of food intake. The present results add to and extend previous findings by suggesting that fruit and vegetable consumption has immediate beneficial psychological effects. Overall, of 14 different main food categories, vegetables consumption contributed the largest share to eating happiness measured across eight days. Thus, in addition to the investment in future well-being indicated by previous research^8^, “healthy” food choices seem to be an investment in the in-the moment well-being.

Importantly, although many cultures convey the belief that eating certain foods has a greater hedonic and mood boosting effect, the present results suggest that this might not reflect actual in-the-moment experiences accurately. Even though people often have a spontaneous “unhealthy = tasty” intuition^13^, thus indicating that a stronger happiness boosting effect of “unhealthy” food is to be expected, the induced eating happiness of sweets did not differ on average from “healthy” food choices such as fruits or vegetables. This was also true for other stereotypically “unhealthy” foods such as pastries and salty extras, which did not show the expected greater boosting effect on happiness. Moreover, analyses on the meal type level support this notion, since snacks, despite their overall positive effect, were not the most psychologically beneficial meal type, i.e., dinner had a comparable “happiness” signature to snacking. Taken together, “healthy choices” seem to be also “happy choices” and at least comparable to or even higher in their hedonic value as compared to stereotypical “unhealthy” food choices.

In general, eating happiness was high, which concurs with previous research from field studies with generally healthy participants. De Castro, Bellisle, and Dalix^22^ examined weekly food diaries from 54 French subjects and found that most of the meals were rated as appealing. Also, the observed differences in average eating happiness for the 14 different food categories, albeit statistically significant, were comparable small. One could argue that this simply indicates that participants avoided selecting bad food^22^. Alternatively, this might suggest that the type of food or food categories are less decisive for experienced eating happiness than often assumed. This relates to recent findings in the field of comfort and emotional eating. Many people believe that specific types of food have greater comforting value. Also in research, the foods eaten as response to negative emotional strain, are typically characterised as being high-caloric because such foods are assumed to provide immediate psycho-physical benefits^18^. However, comparing different food types did not provide evidence for the notion that they differed in their provided comfort; rather, eating in general led to significant improvements in mood^19^. This is mirrored in the present findings. Comparing the eating happiness of “healthy” food choices such as fruits and vegetables to that of “unhealthy” food choices such as sweets shows remarkably similar patterns as, on average, they were associated with high eating happiness and their range of experiences ranged from very negative to very positive.

This raises the question of why the idea that we can eat indulgent food to compensate for life’s mishaps is so prevailing. In an innovative experimental study, Adriaanse, Prinsen, de Witt Huberts, de Ridder, and Evers^23^ led participants believe that they overate. Those who characterised themselves as emotional eaters falsely attributed their over-consumption to negative emotions, demonstrating a “confabulation”-effect. This indicates that people might have restricted self-knowledge and that recalled eating episodes suffer from systematic recall biases^24^. Moreover, Boelsma, Brink, Stafleu, and Hendriks^25^ examined postprandial subjective wellness and objective parameters (e.g., ghrelin, insulin, glucose) after standardised breakfast intakes and did not find direct correlations. This suggests that the impact of different food categories on wellness might not be directly related to biological effects but rather due to conditioning as food is often paired with other positive experienced situations (e.g., social interactions) or to placebo effects^18^. Moreover, experimental and field studies indicate that not only negative, but also positive, emotions trigger eating^15,26^. One may speculate that selective attention might contribute to the “myth” of comfort food^19^ in that people attend to the consumption effect of “comfort” food in negative situation but neglect the effect in positive ones.

The present data also show that eating behaviour in the real world is a complex behaviour with many different aspects. People make more than 200 food decisions a day^27^ which poses a great challenge for the measurement of eating behaviour. Studies often assess specific food categories such as fruit and vegetable consumption using Food Frequency Questionnaires, which has clear advantages in terms of cost-effectiveness. However, focusing on selective aspects of eating and food choices might provide only a selective part of the picture^15,17,22^. It is important to note that focusing solely on the “unhealthy” food choices such as sweets would have led to the conclusion that they have a high “indulgent” value. To be able to draw conclusions about which foods make people happy, the relation of different food categories needs to be considered. The more comprehensive view, considering the whole dietary behaviour across eating occasions, reveals that “healthy” food choices actually contributed the biggest share to the total experienced eating happiness. Thus, for a more comprehensive understanding of how eating behaviours are regulated, more complete and sensitive measures of the behaviour are necessary. Developments in mobile technologies hold great promise for feasible dietary assessment based on image-assisted methods^28^.

As fruits and vegetables evoked high in-the-moment happiness experiences, one could speculate that these cumulate and have spill-over effects on subsequent general well-being, including life satisfaction across time. Combing in-the-moment measures with longitudinal perspectives might be a promising avenue for future studies for understanding the pathways from eating certain food types to subjective well-being. In the literature different pathways are discussed, including physiological and biochemical aspects of specific food elements or nutrients^7^.

The present EMA based data also revealed that eating happiness varied greatly within the 14 food categories and meal types. As within food category variance represented more than two third of the total observed variance, happiness varied according to nutritional characteristics and meal type; however, a myriad of factors present in the natural environment can affect each and every meal. Thus, widening the “nourishment” perspective by including how much, when, where, how long, and with whom people eat might tell us more about experienced eating happiness. Again, mobile, in-the-moment assessment opens the possibility of assessing the behavioural signature of eating in real life. Moreover, individual factors such as eating motives, habitual eating styles, convenience, and social norms are likely to contribute to eating happiness variance^5,29^.

A key strength of this study is that it was the first to examine experienced eating happiness in non-clinical participants using EMA technology and imagery to assess food intake. Despite this strength, there are some limitations to this study that affect the interpretation of the results. In the present study, eating happiness was examined on a food based level. This neglects differences on the individual level and might be examined in future multilevel studies. Furthermore, as a main aim of this study was to assess real life eating behaviour, the “natural” observation level is the meal, the psychological/ecological unit of eating^30^, rather than food categories or nutrients. Therefore, we cannot exclude that specific food categories may have had a comparably higher impact on the experienced happiness of the whole meal. Sample size and therefore Type I and Type II error rates are of concern. Although the total number of observations was higher than in previous studies (see for example, Boushey *et al*.^28^ for a review), the number of participants was small but comparable to previous studies in this field^20,31–33^. Small sample sizes can increase error rates because the number of persons is more decisive than the number of nested observations^34^. Specially, nested data can seriously increase Type I error rates, which is rather unlikely to be the case in the present study. Concerning Type II error rates, Aarts *et al*.^35^ illustrated for lower ICCs that adding extra observations per participant also increases power, particularly in the lower observation range. Considering the ICC and the number of observations per participant, one could argue that the power in the present study is likely to be sufficient to render the observed null-differences meaningful. Finally, the predominately white and well-educated sample does limit the degree to which the results can be generalised to the wider community; these results warrant replication with a more representative sample.

Despite these limitations, we think that our study has implications for both theory and practice. The cumulative evidence of psychological benefits from healthy food choices might offer new perspectives for health promotion and public-policy programs^8^. Making people aware of the “healthy = happy” association supported by empirical evidence provides a distinct and novel perspective to the prevailing “unhealthy = tasty” folk intuition and could foster eating choices that increase both in-the-moment happiness and future well-being. Furthermore, the present research lends support to the advocated paradigm shift from “food as health” to “food as well-being” which entails a supporting and encouraging rather constraining and limiting view on eating behaviour.

## Methods

The study conformed with the Declaration of Helsinki. All study protocols were approved by University of Konstanz’s Institutional Review Board and were conducted in accordance with guidelines and regulations. Upon arrival, all participants signed a written informed consent.

### Participants

Thirty-eight participants (28 females: average age = 24.47, *SD* = 5.88, range = 18–48 years) from the University of Konstanz assessed their eating behaviour in close to real time and in their natural environment using an event-based ambulatory assessment method (EMA). No participant dropped out or had to be excluded. Thirty-three participants were students, with 52.6% studying psychology. As compensation, participants could choose between taking part in a lottery (4 × 25€) or receiving course credits (2 hours).

### Procedure

Participants were recruited through leaflets distributed at the university and postings on Facebook groups. Prior to participation, all participants gave written informed consent. Participants were invited to the laboratory for individual introductory sessions. During this first session, participants installed the application movisensXS (version 0.8.4203) on their own smartphones and downloaded the study survey (movisensXS Library v4065). In addition, they completed a short baseline questionnaire, including demographic variables like age, gender, education, and eating principles. Participants were instructed to log every eating occasion immediately before eating by using the smartphone to indicate the type of meal, take pictures of the food, and describe its main components using a free input field. Fluid intake was not assessed. Participants were asked to record their food intake on eight consecutive days. After finishing the study, participants were invited back to the laboratory for individual final interviews.

### Measures

Immediately before eating participants were asked to indicate the type of meal with the following five options: breakfast, lunch, afternoon tea, dinner, snack. In Germany, “afternoon tea” is called “Kaffee & Kuchen” which directly translates as “coffee & cake”. It is similar to the idea of a traditional “afternoon tea” meal in UK. Specifically, in Germany, people have “Kaffee & Kuchen” in the afternoon (between 4–5 pm) and typically coffee (or tea) is served with some cake or cookies. Dinner in Germany is a main meal with mainly savoury food.

After each meal, participants were asked to rate their meal on three dimensions. They rated (1) how much they enjoyed the meal, (2) how pleased they were with their meal, and (3) how tasty their meal was. Ratings were given on a scale of one to 100. For reliability analysis, Cronbach’s Alpha was calculated to assess the internal consistency of the three items. Overall Cronbach’s alpha was calculated with α = 0.87. In addition, the average of the 38 Cronbach’s alpha scores calculated at the person level also yielded a satisfactory value with α = 0.83 (*SD* = 0.24). Thirty-two of 38 participants showed a Cronbach’s alpha value above 0.70 (range = 0.42–0.97). An overall score of experienced happiness of eating was computed using the average of the three questions concerning the meals’ enjoyment, pleasure, and tastiness.

### Analytical procedure

The food pictures and descriptions of their main components provided by the participants were subsequently coded by independent and trained raters. Following a standardised manual, additional components displayed in the picture were added to the description by the raters. All consumed foods were categorised into 14 different food categories (see Table 1) derived from the food classification system designed by the German Nutrition Society (DGE) and based on the existing food categories of the German Nutrient Database (Max Rubner Institut). Liquid intake and preparation method were not assessed. Therefore, fats and additional recipe ingredients were not included in further analyses, because they do not represent main elements of food intake. Further, salty extras were added to the categorisation.

No participant dropped out or had to be excluded due to high missing rates. Missing values were below 5% for all variables. The compliance rate at the meal level cannot be directly assessed since the numbers of meals and snacks can vary between as well as within persons (between days). As a rough compliance estimate, the numbers of meals that are expected from a “normative” perspective during the eight observation days can be used as a comparison standard (8 x breakfast, 8 × lunch, 8 × dinner = 24 meals). On average, the participants reported *M* = 6.3 breakfasts (*SD* = 2.3), *M* = 5.3 lunches (*SD* = 1.8), and *M* = 6.5 dinners (*SD* = 2.0). In comparison to the “normative” expected 24 meals, these numbers indicate a good compliance (approx. 75%) with a tendency to miss six meals during the study period (approx. 25%). However, the “normative” expected 24 meals for the study period might be too high since participants might also have skipped meals (e.g. breakfast). Also, the present compliance rates are comparable to other studies. For example, Elliston *et al*.^36^ recorded 3.3 meal/snack reports per day in an Australian adult sample and Casperson *et al*.^37^ recorded 2.2 meal reports per day in a sample of adolescents. In the present study, on average, *M* = 3.4 (*SD* = 1.35) meals or snacks were reported per day. These data indicate overall a satisfactory compliance rate and did not indicate selective reporting of certain food items.

To graphically visualise data, Tableau (version 10.1) was used and for further statistical analyses, IBM SPSS Statistics (version 24 for Windows).

### Data availability

The dataset generated and analysed during the current study is available from the corresponding authors on reasonable request.

## Electronic supplementary material

Supplementary Table S1
