# Supplementary material for: Healthy food choices are happy food choices: Evidence from a real life sample using smartphone based assessments
Source: Sci Rep. 2017 Dec 6;7:17069. doi: 10.1038/s41598-017-17262-9 (PMC5719018; doi:10.1038/s41598-017-17262-9)
Supplement: Supplementary file 1 — Supplementary Table S1 [file 41598_2017_17262_MOESM1_ESM.pdf]

Healthy food choices are happy food choices:

Evidence from a real life sample using smartphone based assessments

Deborah R. Wahl[§*], Karoline Villinger[§], Laura M. König,
Katrin Ziesemer, Harald T. Schupp & Britta Renner*

Department of Psychology, University of Konstanz

§ Both authors contributed equally to this work.

* Corresponding authors:
Deborah Wahl
Department of Psychology
University of Konstanz
P.O. Box 47
78457 Konstanz
Germany
Email: deborah.wahl@uni-konstanz.de

Britta Renner
Department of Psychology
University of Konstanz
P.O. Box 47
78457 Konstanz
Germany
Email: britta.renner@uni-konstanz.de

Supplementary Information

Table S1: Descriptive statistics for eating happiness for lunch and dinner by food category.

	Lunch			Dinner		
**Food category**	***N***	***M (SD)***	***Sum***	***N***	***M (SD)***	***Sum***
Vegetables	140	73.88 (19.08)	10,343	180	81.49 (14.71)	14,669
Fruits	19	80.44 (14.56)	1,528	24	84.69 (16.03)	2,033
Sweets	19	75.72 (18.63)	1,439	9	81.11 (17.14)	730
Salty extras	*NA*	*NA*	*NA*	*NA*	*NA*	*NA*
Pastries	*NA*	*NA*	*NA*	1	22.67	23
Bread	49	73.28 (18.38)	3,591	78	80.77 (13.49)	6,300
Pasta	85	73.4 (18.51)	6,239	84	81.06 (14.32)	6,809
Cereals	25	70.15 (20.35)	1,754	19	82.88 (14.66)	1,575
Potatoes	32	75.66 (22.39)	2,421	23	87.17 (10.27)	2,005
Dairy products	75	70.02 (18.96)	5,251	117	80.40 (14.09)	9,407
Meat	71	76.12 (17.11)	5,405	70	80.82 (15.53)	5,657
Eggs	4	62.00 (22.86)	248	14	82.24 (11.78)	1,151
Meat substitutes	7	86.09 (11.01)	603	13	82.28 (12.13)	1,070
Fish	14	68.98 (21.55)	966	7	76.48 (12.57)	535
Total	203	73.09 (18.99)	14,838	245	81.47 (14.73)	19,959

Note: Eating happiness ranged from 1 (low) to 100 (high). Within meal type, multiple selection of food categories was possible. *NA* = no data available.
